# A comparison of the welfare of free-ranging native pony herds on common land with those used for conservation grazing in the UK

**DOI:** 10.1017/awf.2024.35

**Published:** 2024-09-16

**Authors:** Sophia McDonald, Jessica J Harley, Jo Hockenhull

**Affiliations:** 1 Bristol Veterinary School, University of Bristol, Bristol BS40 5DU, UK; 2 University Centre Reaseheath, Rease Heath, Nantwich CW5 6DF, UK; 3 Knowsley Safari, Knowsley, Prescot L34 4AN, UK

**Keywords:** animal welfare, Dartmoor, equine, Exmoor, extensive grazing, welfare assessment

## Abstract

Free-ranging native Dartmoor and Exmoor ponies have not only held strong cultural and environmental significance for thousands of years within their respective national parks, but their environmental benefits and naturally selected characteristics have also been acknowledged and harnessed for conservation grazing and rewilding programmes. Despite a wealth of literature regarding the welfare of sports, leisure and working horses, there is little information concerning the welfare of free-ranging and extensively grazing ponies. The present study compared the welfare of native Exmoor and Dartmoor ponies grazing on the moors in their respective national parks (n = 47) with those that have been translocated to other areas of the UK for use in conservation grazing and rewilding programmes (n = 29) using a specifically designed observational welfare assessment protocol for free-ranging ponies. The results showed a significant difference between common land and conservation grazing ponies in the scores for Body Condition Score, Water Quality and Availability, Environmental Hazards, Human Disturbance, Skin and Coat Condition and the Human Approach Test. Despite no evidence of significant welfare compromise being identified, this study emphasises the importance of year-round monitoring of welfare and the feasibility of the observational welfare protocol to be used by pony keepers and grazing managers in the future.

## Introduction

The majority of owned domesticated horses (*Equus caballus*) in the UK are kept within an intensive husbandry and management system, and only a minority are managed extensively and considered free ranging (Longland 2013; Gonçalves Abrantes 2021). Dartmoor (945 km^2^) and Exmoor (693 km^2^) National Parks in southern England have been populated with free-ranging, semi-feral ponies for thousands of years (Green 2013), and today herds of native Dartmoor and Exmoor ponies are grazed on the common land within the respective national parks. Extensively grazing herds are free to forage, browse and move freely with conspecifics (Christensen *et al*. 2002; King *et al*. 2013; Viksten 2016). Being able to exhibit these natural behaviours means a reduced incidence of health issues (Yngvesson *et al*. 2019), possibly denoting a more positive welfare state compared to stabled horses whose movement is restricted and access to both forage and conspecifics may be restricted (Hartmann *et al*. 2012). This has potentially contributed to the lack of research into the welfare of free-ranging horses, even though this environment does not eliminate the potential for welfare issues to arise (Fraser 2008, 2009).

The genomes of native pony breeds have been shaped by natural selection, meaning that they possess adaptations that are now absent in domesticated breeds (Fraser *et al*. 2019). Native ponies are particularly well-suited to extensive grazing as their hardy nature means they are able to tolerate environmental conditions that other domesticated equines would not be well adapted to (Hodder *et al*. 2005). However, both the Dartmoor and Exmoor breeds are considered priority equine breeds (Rare Breeds Survival Trust 2022) and UK Native Breeds at Risk. Appropriate management is required to carefully increase the number of registered ponies whilst maintaining genetic diversity (Green 2013). Furthermore, their longstanding presence has created a strong cultural heritage, and many stakeholders believe declining numbers are impacting biodiversity within their grazing areas (Udall *et al*. 2023). Low-intensity grazing is beneficial across the trophic levels in upland areas in the UK (Evans *et al*. 2015), where light grazing of swards can increase overall foliage growth (Bott *et al*. 2013), which increases the number of ecological niches, as well as the habitat quality (Kruess & Tscharntke 2002; Wallis de Vries *et al*. 2007; Bussan 2022). These benefits have been recognised as a conservation tool to reduce the presence of invasive species and promote native and/or high-value species (Fraser *et al*. 2019). Therefore free-ranging native ponies provide an attractive choice for use as conservation grazers (Grazing Animals Project 2008) and in rewilding programmes throughout the UK and Europe (Linnartz & Meissner 2014).

UK equines are protected under the Animal Welfare Act (2006) and the additional Code of Practice for the Welfare of Horses, Ponies, Donkeys and Their Hybrids (DEFRA 2017), but this legislation is problematic to apply to free-ranging ponies. Traditionally, ponies are gathered off the moors and onto farms in the autumn, referred to as ‘drifts’, each pony is checked, with some selectively removed and sold in order to manage the population size (Van de Weerd *et al*. 2012). Despite being in place to assess welfare, ‘drifts’ have been highlighted as a specific welfare issue for free-ranging ponies, alongside overbreeding and lack of supplementary food in winter, as gatherings are considered to be stressful (Horseman *et al*. 2016), likely due to infrequent human handling (Jezierski *et al*. 1999). There is also a responsibility to ensure the welfare of ponies acquired for use in conservation grazing and rewilding schemes. Lack of knowledge and poor advice-seeking behaviour have been identified as the root causes of equine welfare issues in the UK (Horseman *et al*. 2016) and there are anecdotal concerns that those responsible for conservation grazing ponies are unfamiliar with the species or lack prior experience. Although marketed at these programmes for their hardiness and ability to adapt (Grazing Animals Project 2001); ponies are grazing in an unfamiliar environment and with altered social group dynamics which is likely to increase stress levels (van Dierendonck 2006), and so has the potential to negatively impact their welfare. A lack of awareness could compromise individual welfare and potentially that of the wider equine community, for example if there was an unrecognised infectious disease outbreak.

Continued criticism of the management of free-ranging ponies by both welfare organisations and the public (Harley *et al*. 2021; Udall *et al*. 2023), means that necessary action must be taken to adapt present management systems to secure the future of these ponies (Green 2013). Whilst population-level management (for a discussion, see Green 2013; Petrie-Ritchie 2015), is important to maximise herd-level welfare; it is also imperative to monitor welfare at an individual level to identify issues and potential risk factors to allow for early intervention and safeguarding of pony welfare (Blokhuis et al. 2010). Welfare assessment protocols for sport, leisure and working horses, of which there are several (e.g. AWIN 2015; Dalla Costa *et al*. 2016), often refer to free-ranging horses’ physical and social environments as natural and welfare-friendly conditions (Waran 1997). This has impacted the design and utilisation of suitable welfare assessment protocols for free-ranging ponies (Harley *et al*. 2021). Despite the recent development of a few specific protocols (e.g. Harvey *et al*. 2020; Harley *et al*. 2021), free-ranging native pony welfare in the UK is under-researched, with no known published studies attempting to report upon individual welfare or make comparisons between populations. This study aims to compare the welfare of free-ranging native pony herds grazing on the common land within Dartmoor and Exmoor National Parks, with herds that have been translocated to other areas for the specific purpose of conservation grazing or rewilding, using an adapted version of the welfare assessment designed by Harley *et al*. (2021).

## Materials and methods

### Ethical approval

This study was granted ethical approval by the University of Bristol’s Animal Welfare and Ethical Review Body on 2nd May 2023; unique reference number UIN/23/017.

### Study sites and populations

Six study sites, accessible to the public and with a population of free-ranging Dartmoor or Exmoor ponies, were selected (Table 1). These sites hosted registered Exmoor ponies or heritage/registered Dartmoor ponies. Ponies assessed were chosen opportunistically from the free-ranging population during the first visit to each location.

**Table 1.** Details of free-ranging native pony herds including location, breed, sexes, ages, and numbers of ponies assessed

Herd location and range size	National Grid Reference	Group	Breed	Number of ponies assessed	Sexes	Ages
Haddon Hill 1.9 km^2^	SS800383	Common Land	Exmoor	11	10 mares 1 stallion	9 adults 2 sub-adults
Winsford Hill 6.2 km^2^	SS899340	Common Land	Exmoor	12	11 mares 1 colt	11 adults 1 foal
Widecombe Hill 1.9 km^2^	SX716768	Common Land	Dartmoor	11	10 mares 1 stallion	10 adults 1 foal
Bellever Tor 2.6 km^2^	SX641771	Common Land	Dartmoor	13	11 mares 2 geldings	13 adults
Knepp Castle Estate 2.8 km^2^	TQ154240	Conservation Grazing	Exmoor	15	12 mares Foal sexes undetermined	11 mares 1 sub-adult 3 foals
Grimston Warren 0.69 km^2^	TF665226	Conservation Grazing	Dartmoor	14	7 mares 7 geldings	14 adults

### Data collection

All individual welfare assessments were carried out between 0800 and 1700h from 30th May to 24th July 2023 by the first author (SM) who has extensive background knowledge of equine care and management. Weather conditions, assessment time and necessary assessment data were recorded. A minimum of 3 m was always maintained between the pony and assessor and binoculars were used if necessary. Photographic and written descriptions allowed individual pony identification in subsequent visits, including details of GPS location, sex, colour, markings, and branding/other mutilations (if present).

The welfare assessment protocol comprises 15 animal- and resource-based indicators (fully detailed in Table 2). Indicators are scored on a numerical categorical scale from zero to three. Zero or one indicates a negative state, two is neutral and some indicators can be scored with three representing a positive state. Only specific indicators can be assigned a score of zero (see Table 3), which indicates a more serious welfare compromise. If any pony received a score of zero and was deemed to have seriously compromised welfare, the Horse Grimace Scale (HGS) (developed by Dalla Costa *et al*. 2014a) was used and scored between zero to two. The issue was reported to the relevant welfare authority responsible for that individual.

**Table 2.** Welfare assessment protocol (adapted from Harley *et al*., 2021) with details of animal- (AB) and resource-based (RB) indicators and scoring categories for each welfare indicator

Score category	0	1	2	3
**Welfare indicator**				
**Body Condition Score** (AB) using 9–point scale (Henneke *et al*., 1983)	Poor/very thin (1–2) *or* fat/extremely fat (8–9).	Thin/moderately thin (3–4).	Moderate to fleshy (5–7).	–
**Water Quality and** **Availability** (RB)	–	No water detected or is inaccessible.	Access to stagnant pooling water, puddle, or marsh area.	Access to fresh, free running body of water.
**Human Disturbance** (RB) (presence of walkers, dogs, cyclists)	–	> 20	1 – 20	None.
**Environment Hazards** (RB) (human–made hazards, e.g. fencing material, wire, refuse)	–	Hazards prevent the pony’s ability to move freely.	Hazards are present but can be avoided by the pony.	No hazards present.
**Resting Comfort** (RB)	–	No clean, dry area to lay down or rest, there are > 10 people present.	Clean, dry area to lay down or rest, there are < 10 people present.	Clean, dry area to lay down or rest with no human disturbance.
**Thermal Environment** (RB) (access to shelter/shade, e.g. stone wall, trees, hedges) **and Comfort** (AB) (shivering or sweating)	–	No access to shelter or shade *and/or* is shivering or sweating.	No access to shelter or shade *and* is not shivering or sweating.	Access to shelter and/or shade *and* is not shivering or sweating.
**Mobility** (AB)	Immobile or severely compromised mobility, where the pony cannot keep up with the herd.	Walking with abnormality, uneven weight–bearing but remains with the herd.	No signs of abnormality, evenly bearing weight on all limbs whilst standing and walking.	–
**Mane and Tail Condition** (AB)	–	Hair is missing or broken in the mane or tail.	Normal tail and mane with no hair loss.	–
**Skin and Coat** **Condition** (AB) (excluding mane and tail)	–	Coat is patchy or uneven, skin may be visible (alopecia) in places.	Coat appears healthy and in good condition.	–
**Ocular Discharge** (AB)	Discharge and partial or full closure, with or without swelling.	Discharge with eye open (mucus in the eye and discharge down cheek).	Normal eye with no discharge.	–
**Nasal Discharge** (AB)	–	Nasal discharge present.	No nasal discharge.	–
**Coughing** (AB)	–	Pony is coughing persistently.	No coughing observed.	–
**Wounds and Swelling** (AB)	Acute, open wound (> 7 cm) present involving deeper tissue, infection present.	Wound (< 7 cm), appears to be healing *or* visible swelling to a skin area < 7 cm with or without hair loss.	No wounds or swollen areas of skin.	–
**Social Contact** (RB)	–	Solitary (no other ponies visible).	Conspecifics present.	–
**Human Approach Test** (AB)		Moves away when assessor is > 9 m away.	Moves away when assessor is < 9 m away.	Does not move away from assessor (must stop at 3 m).
***Horse Grimace Scale*** *(AB)* *(Only applied if zero is scored for any indicator)*	HGS score 8–12	HGS score 4–7	HGS score 0–3

**Table 3.** Score frequencies for common land (n = 47) and conservation grazing ponies (n = 29), with the results of Mann Whitney *U* tests between the two groups for each of the animal- and resource-based welfare indicators that make up the welfare assessment (adapted from Harley *et al*. 2021). Significant differences between the two groups are represented with bold P-values

Welfare indicator	Score	Common Land†	Conservation Grazing†	W	*P*-value*
Body Condition Score	**0**		3	752.0	**0.026**
	**1**				
	**2**	47	26		
Water Quality and Availability	**1**	36	14	488.5	**0.012**
	**2**				
	**3**	11	15		
Human Disturbance	**1**			1247	**< 0.001**
	**2**	8	29		
	**3**	39			
Environmental Hazards	**1**			565.5	**0.020**
	**2**	8			
	**3**	39	29		
Resting Comfort	**1**			681.5	N/A
	**2**				
	**3**	47	29		
Thermal Environment and Comfort	**1**			681.5	N/A
	**2**				
	**3**	47	29		
Mobility	**0**			681.5	N/A
	**1**				
	**2**	47	29		
Mane and Tail Condition	**1**	1		667.0	0.45
	**2**	46	29		
Skin and Coat Condition	**1**	6		594.5	**0.048**
	**2**	41	29		
Ocular Discharge	**0**			681.5	N/A
	**1**				
	**2**	47	29		
Nasal Discharge	**1**			681.5	N/A
	**2**	47	29		
Coughing	**1**			681.5	N/A
	**2**	47	29		
Wounds and Swelling	**0**			676.0	0.87
	**1**	2	1		
	**2**	45	28		
Social Contact	**1**			681.5	N/A
	**2**	47	29		
Human Approach Test	**1**	1		917.0	**0.0023**
	**2**	24	26		
	**3**	22	3		
*Horse Grimace Scale*	**0**			–	–
	**1**				
	**2**				

* N/A indicates that the statistical test could not be performed due to no data variation for that indicator (i.e. all ponies had the same score).

† where there is no value, n = 0 for that score.

A Qualitative Behavioural Assessment (QBA), initially developed by Wemelsfelder et al. (2000, 2001) was also carried out. The protocol conducted for the QBA required the assessor to spend two to three minutes familiarising themselves with the pony’s demeanour (expressive behaviour) followed by a 10 minute focal observation (Altmann 1974). Scores were entered after the observation period concluded. Descriptors were selected to represent the expressive qualities of a free-ranging pony and chosen from current equine studies applying QBA to evaluate horses’ behavioural responsiveness (AWIN 2015; Hintze *et al*. 2017; Czycholl et al. 2019). QBA descriptors were initially trialled on 13 individual Carneddau Mountain ponies by one of the authors (JJH), and a specialist equine surgeon. The initial trial resulted in a moderate degree of reliability between assessors for the descriptor ‘friendly’, all other descriptors were good or very good. Prior to inclusion in our study, the definition and description were refined for better context, i.e. ‘friendly’ was changed to Friendly (Sociable) and the definition was expanded to ensure the situation of the behaviour was limited to the pony’s interaction with other pony(s) rather than the assessor (Table 4).

**Table 4.** List of Qualitative Behavioural Assessment descriptors and definitions (modified from Fleming *et al*. 2013; Dalla Costa *et al*. 2016; Hintze et al. 2017)

Term	Description of behavioural term
Agitated	Not settling, fidgety, annoyed, tail swishing, ears flicking, stamping leg, shaking head.
Curious	Exploring, inquisitive, desire to investigate environment or another animal/pony.
Fearful	Startled, afraid, hesitant, uneasy, tense.
Relaxed	Not tense or rigid, peaceful/tranquil, content.
Apathetic	Showing little or no emotion, depressed, showing no interest in environment or ponies around them.
Friendly (Sociable)	Affectionate, companionable, actively engages with another pony(s).
Withdrawn	Unsociable, shy, reclusive, not searching for contact with others, solitary (may be within the area of group but not engaging with group).
Alert	Ears moving around, can hear something of interest, may lift head toward area of interest.
Aggressive	Hostile, attacking, dominant, angry.
Responsive	Active, receptive, aware of environment, responding to what is happening in environment.

All locations were revisited within 21 days of the first assessment and the welfare assessment was repeated on individuals that could be successfully re-identified (n = 41) to test for assessor reliability.

### Statistical analysis

All analyses were performed in the R-4.2.2 (R-Studio Team 2021). Datasets were checked for violations of the assumptions of normality (normality of residuals and homogeneity of variance), data transformations were attempted, and a non-parametric test was used if unsuccessful. The threshold for significance in all statistical tests was *P* < 0.05.

### Comparing common land and conservation grazing ponies

To investigate if there was a statistically significant difference between each welfare indicator (Table 3) for common land and conservation grazing ponies, Mann-Whitney *U* tests were performed. To investigate if there was a statistically significant difference between each welfare indicator for Dartmoor and Exmoor breeds grazing on both land types (Dartmoor ponies grazing on common land [CLDM], Exmoor ponies grazing on common land [CLEX], Dartmoor ponies in conservation grazing programmes [CGDM] and Exmoor ponies in conservation grazing programmes [CGEX]), a Kruskal-Wallis test was used. Pair-wise Wilcoxon Rank Sum Tests were calculated with a Benjamini-Hochberg correction for multiple testing to identify groups with significant differences.

### Qualitative Behavioural Assessment

To analyse the QBA scores, a Principal Component Analysis (PCA, correlation matrix, no rotation) was conducted separately for common land and conservation grazing ponies. A PCA was used to reduce the number of dimensions to assist in explaining the majority of variation between the individual ponies.

### Test/retest reliability

To investigate assessor reliability, test/retest analysis was undertaken using Cohen’s unweighted kappa test statistic and the percentage of agreement between visits for each welfare indicator (as used by Harley *et al*. 2021). Kappa values were interpreted according to the work of Altman (1991), where values of 0.81–1.00 are considered very good, 0.61–0.80 are considered good, 0.41–0.60 are considered moderate, 0.21–0.40 are considered fair, and < 0.20 are considered poor agreement.

## Results

A total of 76 ponies underwent assessment, 38 individuals of each breed (Dartmoor and Exmoor), 47 on the common land and 29 within conservation grazing/rewilding programmes (Table 1).

The Horse Grimace Scale (see Table 2) was not undertaken for any individual as no pony was deemed to have seriously compromised welfare at the time the welfare assessment was conducted.

### Comparing common land and conservation grazing ponies

The scores for Body Condition Score (BCS), Human Disturbance, Environmental Hazards and Skin and Coat Condition were significantly lower for conservation grazing ponies compared to common land ponies (Table 3). Scores for Water Quality and Availability, and the Human Approach Test were significantly higher for common land ponies compared to conservation grazing ponies (Table 3). There was no significant difference between the scores of the two groups for Resting Comfort, Thermal Environment and Comfort, Mobility, Mane and Tail Condition, Ocular Discharge, Nasal Discharge, Coughing, Wounds and Swelling, and Social Contact (Table 3).

There was a statistically significant difference between the scores for BCS, Water Quality and Availability, Human Disturbance, Environmental Hazards, and Human Approach Test in the four groups (CLDM, CLEX, CGDM, CGEX) (Table 5). There was no significant difference between the four groups for all other welfare indicators (Table 5). *Post hoc* pair-wise Wilcoxon Tests with Benjamini-Hochberg Correction are displayed in Table 5, where there were significant pair-wise comparisons for the five welfare indicators listed above.

**Table 5.** Kruskal-Wallis rank-sum test results between Exmoor common land (CLEX, n = 23), Dartmoor common land (CLDM, n = 24), Exmoor conservation grazing (CGEX, n = 15) and Dartmoor conservation grazing (CGDM, n = 14) ponies, with post hoc pair-wise Wilcoxon tests to determine the significant differences between the four groups for each welfare indicator

Welfare Indicator	Kruskal-Wallis test			*Post hoc* test P-values					
	X^2^	df	*P*-value*	CLDM vs. CLEX	CLDM vs. CGEX	CLDM vs. CGDM	CLEX vs. CGEX	CLEX vs. CGDM	CGEX vs. CGDM
Body Condition Score	12.5	3	**0.0058**	–	**0.045**	–	**0.045**	–	0.09
Water Quality and Availability	49.8	3	**< 0.001**	**<0.001**	**<0.001**	–	**0.0011**	**0.0024**	**<0.001**
Human Disturbance	54.4	3	**< 0.001**	**<0.001**	**<0.001**	**<0.001**	**<0.001**	**<0.001**	–
Environmental Hazards	20.3	3	**< 0.001**	**0.0054**	–	–	**0.015**	**0.015**	–
Resting Comfort	N/A	3	N/A						
Thermal Environment and Comfort	N/A	3	N/A						
Mobility	N/A	3	N/A						
Mane and Tail Condition	2.30	3	> 0.05	0.47	–	–	0.47	0.47	–
Skin and Coat Condition	5.27	3	> 0.05	0.37	0.36	0.36	0.27	0.27	–
Ocular Discharge	N/A	3	N/A						
Nasal Discharge	N/A	3	N/A						
Coughing	N/A	3	N/A						
Wounds and Swelling	0.87	3	> 0.05	1.00	0.94	0.94	0.94	0.94	0.94
Social Contact	N/A	3	N/A						
Human Approach Test	18.9	3	**< 0.001**	**0.0099**	**0.0042**	**0.0028**	0.62	0.46	0.62

Significant differences between the groups are represented with bold *P* values. *Post hoc* test P-values with “-” present indicate that a comparison between those groups was not available or applicable

* N/A indicates that the statistical test could not be performed due to no data variation for that indicator and therefore *post hoc* pair-wise Wilcoxon tests could not be carried out.

### Qualitative Behavioural Assessment

**Figure 1.**
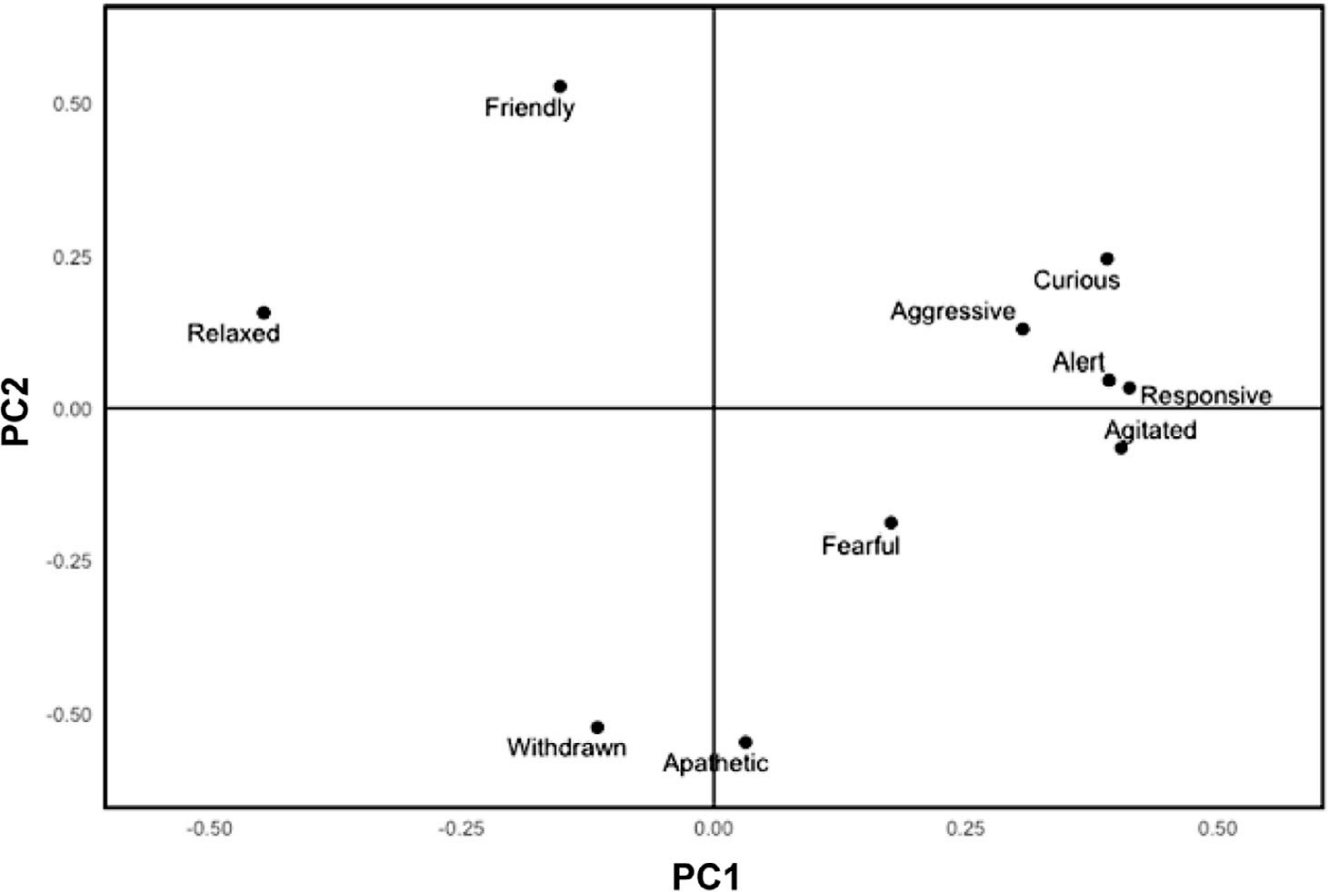
Loadings of the ten Qualitative Behavioural Assessment descriptors along the first two Principal Component Analysis factors (PC1, PC2) for common land (n = 47) ponies.

**Figure 2.**
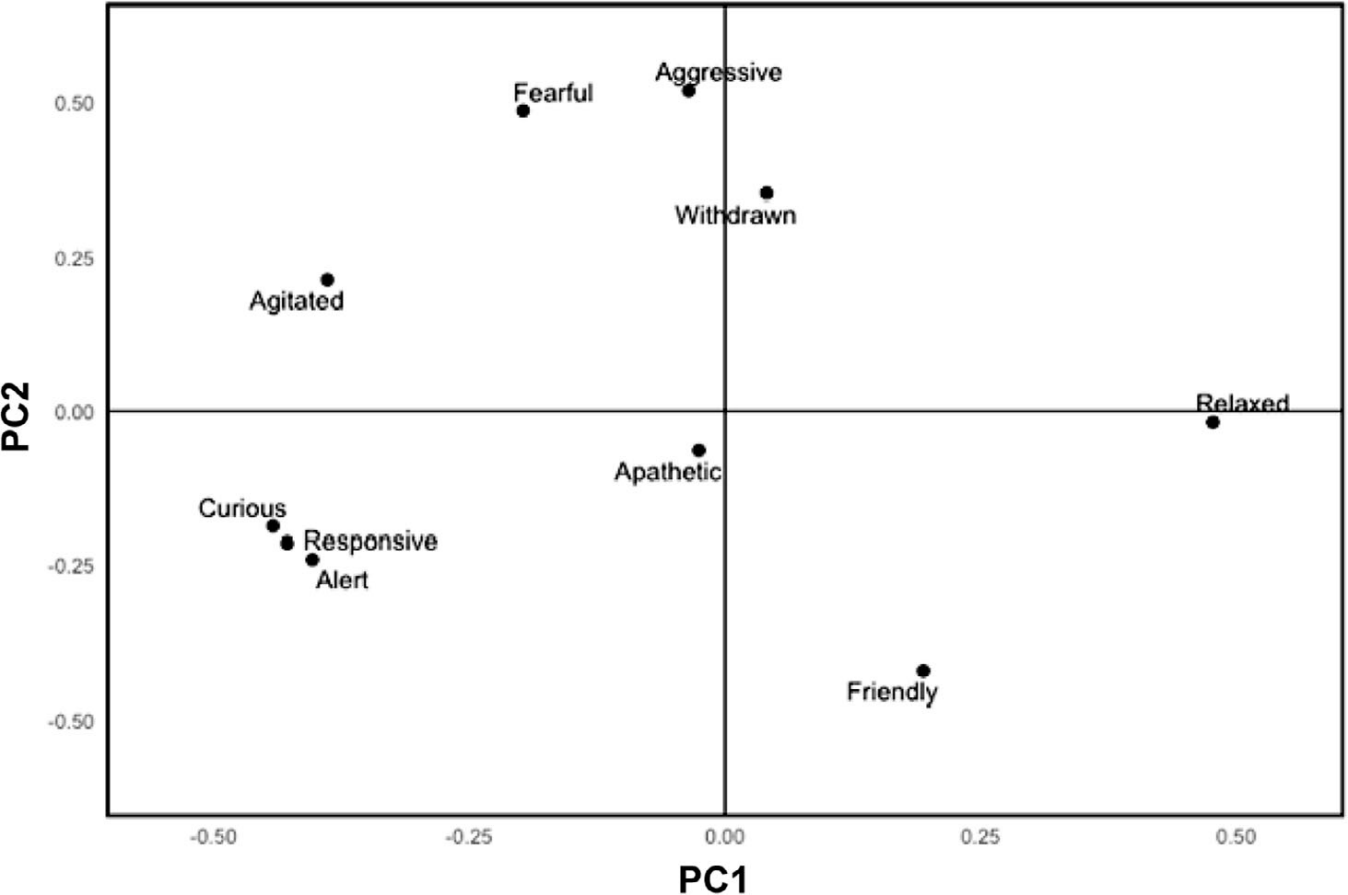
Loadings of Qualitative Behavioural Assessment descriptors along the first two Principal Component Analysis factors (PC1, PC2) for conservation grazing ponies (n = 29).

Figures 1 and 2 show the distribution of the ten descriptors along the first two principal component (PC) factors for common land and conservation grazing ponies, respectively. Many of the terms load strongly on PC1, accounting for 39.4% of the variation in the data for conservation grazing ponies and 38.2% for common land ponies. PC2 accounts for 19.7% of the variance in the data for common land ponies and 14.9% for conservation grazing ponies. PC1 for common land ponies was characterised by terms ranging from ‘relaxed’ to ‘alert/responsive/agitated’, whereas PC2 was described by ‘apathetic/withdrawn’ to ‘friendly’ (Figure 1). PC1 for conservation grazing ponies was characterised by terms ranging from ‘agitated/curious/responsive/alert’ to ‘relaxed’, and PC2 was described by ‘friendly’ to ‘aggressive/fearful’ (Figure 2).

### Test/retest reliability

Some of the welfare indicators scored with 100% agreement between the two assessments and could not be tested for reliability using Cohen’s unweighted kappa test statistic (Table 6). There was very good agreement (kappa estimate of 0.83–1.00) for BCS and Human Disturbance, and moderate agreement (kappa estimate of 0.43) for the Human Approach Test. There was no agreement (kappa estimate of 0) for Water Quality and Availability, Thermal Environment and Comfort, Skin and Coat Condition, Coughing and Social Contact. However, the agreement was greater than 90% for these indicators apart from Water Quality and Availability (Table 6). Environmental Hazards has a kappa estimate of –0.17 suggesting this is due to chance.

**Table 6.** Cohen’s unweighted kappa test statistic results (Kappa estimates, k) and the percentage agreement for each welfare indicator between the two assessments to measure the test/retest reliability

Welfare indicator	Percentage agreement (%)	k*
Body Condition Score	100	1.00
Water Quality and Availability	36.59	0.022
Human Disturbance	92.68	0.83
Environmental Hazards	60.98	–0.17
Resting Comfort	100	N/A
Thermal Environment and Comfort	97.56	0
Mobility	100	N/A
Mane and Tail Condition	100	N/A
Skin and Coat Condition	90.24	0
Ocular Discharge	100	N/A
Nasal Discharge	100	N/A
Coughing	97.56	0
Wounds and Swelling	100	N/A
Social Contact	97.56	0
Human Approach Test	75.61	0.44

* N/A indicates that the statistical test could not be performed due to no data variation for that indicator.

## Discussion

Though there are a number of clear differences in resource-based indicators between common land and conservation grazing ponies, most animal-based indicators showed no significant variation. Animal-based indicators scored positively and consistently suggesting that all surveyed populations were in good general health showing little or no signs of lameness, ocular or nasal discharge, coughing, sweating, shivering, wounds or swelling. Comparable findings have been identified in semi-feral Carneddau Mountain ponies (Harley *et al*. 2021) and horses grazing on ‘parcours’ (outdoor groups-housing system) in France (Dai *et al*. 2023), with infrequent observation of ocular and nasal discharge and coughing. These are more frequent problems in stabled horses (Hotchkiss *et al*. 2007; Visser *et al*. 2014), as respiratory issues are closely linked with indoor housing and have reduced prevalence in the open-air environment of free-ranging ponies.

Although Mane and Tail Condition were consistent across the groups, there was a significant difference in Skin and Coat Condition. Several common land ponies had patchy coats and alopecia (12.7%), which is often attributed to insect irritation causing persistent itching (Górecka & Jezierski 2007; Mullan *et al*. 2014; Dai *et al*. 2023). The welfare assessment was carried out in the summer months on warm, sunny days where insect activity is increased (Strickman *et al*. 1995), providing a likely explanation. Alternatively, bare patches of skin may have been a result of agonistic behaviours (bites or kicks) between mares and sub-adults to express dominance (Sigurjónsdóttir & Haraldsson 2019) or herding behaviours by stallions (Ransom *et al*. 2010), of which there were none present within the surveyed conservation grazing populations and this may be why coats were in better condition in those groups.

BCS differed between common land ponies and CGEX ponies, where some CGEX mares were identified as ‘fat’ (Henneke *et al*. 1983) and scored zero. Upon assessment, these mares scored two, but when the herd was revisited and after discussion with an experienced equine welfare scientist (JoH), some of the mares’ scores were downgraded to zero and therefore HGS was not undertaken at the time of the assessment. As the assessment is solely observational, and there was a potential for these mares to be in foal, correct assessment of BCS was difficult. In comparison to the upland, dry heath habitats of Exmoor and Dartmoor National Park, the ‘Middle Block’ at Knepp Castle Estate is comprised of traditional parkland that was reseeded with grass seed and a local wild meadow seed mix in 2001 (Tree 2018). Horses released in a natural environment within a temperate climate can adapt their daily intake according to pasture availability and changes to climate, maintaining a good BCS (Souris *et al*. 2007) as was the case with all other assessed ponies in the study. However, in the summer, increased appetite and metabolic rate (Arnold *et al*. 2006) have been found to result in fat deposition when high-quality forage is available, and this is likely to be the case here. High BCS has also been identified in other ‘rustic’ breeds (Camargue and Merens) grazing on French ‘parcours’ (Dai *et al*. 2023). Despite the well-reported dangers of a high BCS (e.g. laminitis, colic, insulin resistance), it has been suggested that ponies demonstrate photoperiodically entrained physiological adaptations that promote survival where winter forage is sparse (Fuller *et al*. 2001; Rhind *et al*. 2002; Thiery *et al*. 2002; Henry 2003), and so a higher BCS in the summer months may not have negative repercussions for health or reproduction (Górecka *et al*. 2020), though year-round monitoring would be required to confirm if this is the case here. Furthermore, the use of the cresty neck score (Carter *et al*. 2009) may have been useful as it has been shown to predict insulin dysregulation in ponies (Fitzgerald *et al*. 2019) and therefore may have provided more robust evidence of a welfare compromise than using BCS alone.

CLDM ponies scored significantly higher than the other groups on the Human Approach Test, with most (66.6%) individuals scoring three. Birke *et al*. (2011) identified similar results in flight distances of naïve young Dartmoor ponies, averaging at 2.4 (± 0.54) m in the first session. Reasons for differences between the groups are difficult to determine, as the individual past experiences of each pony are unknown. Some ponies may have been handled more frequently than others, which would have reduced their flight distances over time (as shown by Birke *et al.* 2011) and resulted in more positive scores. Alternatively, others may have had a negative past experience (e.g. aversive handling, stressful transportation), and associate human approach with negative outcomes (Rushen *et al*. 1999; Fureix *et al*. 2012). Greater human presence in conservation grazing areas likely stems from specified public walking routes and designated parking facilities, in contrast to more remote areas in Dartmoor and Exmoor National Parks (e.g. Winsford Hill, Widecombe Hill). Conservation grazing ponies scored significantly lower on the Human Approach Test implying that they would keep a greater distance from the public. However, allowing a closer human approach scored positively in the welfare protocol, despite numerous campaigns to deter the public from feeding or touching free-ranging ponies (e.g. Dartmoor National Park Authority 2017). A close human approach could negatively impact ponies’ welfare as it increases the chances of them being fed or touched, though it may be beneficial if an issue is identified and requires treatment.

Resource-based indicators showed greater variability during revisits compared to animal-based indicators. Despite the variability, resource-based indicators implied that all surveyed populations were in the presence of conspecifics and had access to a suitable resting area with both shade and shelter. Access to shelter is crucial during inclement weather (Tyler 1972; Duncan 1985; Autio & Heiskanen 2005), aiding thermoregulation and prevention of the development of other welfare issues (e.g. shivering, sweating). These indicators relied upon surveying the area within a 500-m circular radius of the observed pony (Harley *et al*. 2021) and were more inconsistent between visits due to differing pony locality. For example, the absence of a water source scored one, implying that the pony may not have access to water. However, nearby water sources may not have been visible or situated differently from where the herd was throughout the assessment, explaining why water quality and availability had a much lower percentage agreement in the test/retest in comparison to the other indicators. Surveying the entire grazing area for water sources and environmental hazards would have reduced subjectivity.

An animal’s affective state moves along a scale of negative and positive valence, influenced by its environment and experience (Mendl *et al*. 2010). Qualitative Behavioural Assessment is a novel, whole-animal method to evaluate an animal’s emotional state and determine its welfare and response to environmental conditions (Wemelsfelder 2007). PC1 in both common land and conservation grazing ponies reflects activity and arousal levels (Figures 1 and 2), although ‘relaxed’ is on opposing ends of the axis. Mullan *et al*. (2014) observed similar findings in tethered and free-ranging horses on public grazing land in South Wales, where PC1 indicated arousal level. PC2 differs between the two groups, where for common land ponies it explains sociability, but for conservation grazing ponies it explains the mood and subjective experience, akin to Mullan *et al*. (2014). QBA improves understanding of individual affective states and has been shown to correlate with other quantitative indicators (Dalla Costa *et al*. 2014b). Statistical analysis is required for QBA interpretation (Dalla Costa *et al*. 2014b), which may reduce its viability if the welfare assessment were to be utilised by pony keepers and stock-person managers. In an app developed by the SRUC (2020) to help perform QBAs, charts are automatically displayed for emotional well-being and facilitate comparison with previous assessments, enhancing accessibility for use by key stakeholders in the future.

Welfare was assessed in the early summer and is only representative of the ponies’ welfare at that time. While sufficient for interpopulation comparisons, year-round assessment is more beneficial to effectively monitor welfare, as free-ranging environments are heavily characterised by significant seasonal variation (Harley *et al*. 2021). Seasons have been shown to significantly affect BCS and the skin condition of extensively grazing Swedish Gotland ponies (Viksten *et al*. 2023). Utilising a similar methodology to that of Viksten *et al*. (2023), assessing welfare every four weeks year-round would enable cross-season comparisons and a better understanding of how each individual adapts to environmental changes (Sterling 2004). The assessment was conducted by one assessor and although the welfare protocol has been previously piloted and tested for reliability (Harley *et al*. 2021), comparing the scores of multiple assessors would have increased reliability. However, re-tests showed a high percentage agreement (> 60%), except for one welfare indicator. Cohen’s unweighted kappa test lacks value and can be misleading when no data variation exists, as the percentage of agreement between the two visits was 100%. The individual welfare state was determined solely from the indicators within the protocol, and being observational, some issues may pass undetected. However, this was the only reasonable approach to take for studying these populations within the given time-frame. Furthermore, the assessment of free-ranging ponies in the field presents the challenge of optimal assessor positioning and distance without disturbing the herd. Sometimes assessment ceased if the herd moved into an unsuitable location (e.g. low, dense trees), reducing sample sizes where all individuals were not able to be sampled.

### Animal welfare implications

The information gathered in this study has provided insight into the welfare of extensively grazing Exmoor and Dartmoor ponies on areas of common land within the respectively named national parks and those enlisted for the benefits of their grazing as part of conservation grazing and rewilding programmes. There is evidence to suggest that conservation grazing locations should be selected carefully and be similar to the ponies’ source habitat; ponies should be monitored closely for the first few weeks when they arrive as conservation grazing areas may have greater levels of human disturbance and increased food availability that could cause health issues (e.g. obesity, laminitis) which would impact welfare. This study also highlights the feasibility of an observational assessment for use by pony keepers and grazing managers to monitor welfare at an individual level and avoid unnecessary stress to ponies by rounding them up (‘drifting’), as well as allowing for more targeted treatment of individuals with issues which will improve herd level welfare.

## Conclusion

Overall, the welfare of Exmoor and Dartmoor ponies grazing on the common land and in conservation grazing/rewilding programmes scores largely similarly, with all ponies assessed as being sound, within a social group and with sufficient access to food and shelter. The constraints of the welfare assessment protocol may have impacted the scores for some resource-based indicators, in particular water quality and availability but with slight adjustment these then may be more representative of the ponies’ grazing area. This study highlights the importance and encourages the monitoring of the welfare of herds of free-ranging native ponies across the UK into the future. It implies that research into the welfare of other free-ranging populations is necessary to secure the future of Exmoor and Dartmoor ponies on the moors which will allow for them to exhibit their benefits as part of conservation grazing and rewilding programmes.
